# Bond Strength—Coordination Number Fluctuation Model of Viscosity: An Alternative Model for the Vogel-Fulcher-Tammann Equation and an Application to Bulk Metallic Glass Forming Liquids

**DOI:** 10.3390/ma3125246

**Published:** 2010-12-10

**Authors:** Masahiro Ikeda, Masaru Aniya

**Affiliations:** 1 Course of General Education, Natural Science, Fukui National College of Technology, Geshi-chou, Sabae, Fukui 916-8507, Japan; 2 Department of Physics, Graduate School of Science and Technology, Kumamoto University, Kumamoto 860-8555, Japan; E-Mail: aniya@gpo.kumamoto-u.ac.jp

**Keywords:** viscosity, VFT equation, BSCNF model, fragility, cooperativity

## Abstract

The Vogel-Fulcher-Tammann (VFT) equation has been used extensively in the analysis of the experimental data of temperature dependence of the viscosity or of the relaxation time in various types of supercooled liquids including metallic glass forming materials. In this article, it is shown that our model of viscosity, the Bond Strength—Coordination Number Fluctuation (BSCNF) model, can be used as an alternative model for the VFT equation. Using the BSCNF model, it was found that when the normalized bond strength and coordination number fluctuations of the structural units are equal, the viscosity behaviors described by both become identical. From this finding, an analytical expression that connects the parameters of the BSCNF model to the ideal glass transition temperature *T*_0_ of the VFT equation is obtained. The physical picture of the Kohlrausch-Williams-Watts relaxation function in the glass forming liquids is also discussed in terms of the cooperativity of the structural units that form the melt. An example of the application of the model is shown for metallic glass forming liquids.

## 1. Introduction

The Vogel-Fulcher-Tammann (VFT) equation [1,2,3] is one of the most commonly used expressions for the analysis of the temperature dependence of viscosity [4,5,6,7,8,9], relaxation time [5,8,10,11], diffusion coefficient [5,6,7,9], and electrical conductivity [5,6,7], *etc*. The application of the VFT equation covers a wide field of research [12]. It has been reported that the transport properties of supercooled melts can change at certain characteristic temperatures such as the dynamical crossover temperature *T*_c_ [8,11,13,14]. By reducing the temperature of the liquid below *T*_c_, the thermally activated hopping process becomes dominant [15]. In such a process, the activation energy for the transport properties is often discussed in the frame of the Arrhenius law expressed as *A* = *A*_0_exp(±*E*_a_/*RT*), where *A*, *A*_0_, *R* and *E*_a_ denote the transport coefficient, its pre-exponential factor, the gas constant, and the activation energy for the transport coefficient, respectively. In this case, the activation energy *E*_a_ is considered as the energy barrier that the mobile species, such as ions or molecules, must overcome to move from one position to another. However, in most cases, the value of *E*_a_ is not a constant [16,17,18] and changes with variation in temperature. Some studies have shown that the variations of the activation energy for the transport properties are affected by their diffusion and structural relaxation mechanism during supercooling [5,8,9,13,16,17,19].

According to the strong-fragile classification of glass forming liquids [13,20,21], systems obeying the Arrhenius law are called strong system. These systems exhibit an almost straight line in the temperature dependence of the viscosity when plotted against the inverse temperature normalized by their glass transition temperature *T*_g_/*T*. Such a plot is usually called Angell’s plot. On the other hand, systems showing a large curvature in the temperature dependence do not follow the Arrhenius law, due to changes of the activation energy *E*_a_ that apparently depends on temperature [16,17,18,19]. Such systems are called fragile system. To describe the behavior observed in fragile systems, the VFT equation has often been employed [4,6,7]. Although the physical background of the VFT equation has been fully discussed from the theoretical point of view [10,22], there might be some discrepancies between the experimental data and its interpretation when the actual data and the VFT equation are compared. The VFT equation has been used as a practical equation to reproduce the experimental data, and therefore it is not sufficient to understand the physics behind the glass forming process. For instance, one of the parameters of the VFT equation, the so-called ideal glass transition temperature *T*_0_ which indicates the dynamical divergence in the temperature dependence of the viscosity or relaxation time, is not observed in real systems [18]. Another point is that the values of the parameters of the VFT equation, obtained from the data analysis, have not been fully exploited. Specifically, although the VFT equation reproduces the experimental data, no concrete microscopic physical picture of the melt and further knowledge on the structural relaxation can be extracted from the VFT parameters alone.

Previously, a model for temperature dependence of the viscosity of melt has been proposed by one of the authors [23]. The model which is called Bond Strength—Coordination Number Fluctuation (BSCNF) model, describes the viscosity behavior in terms of the mean values of the bond strength *E*_0_, the coordination number *Z*_0_, and their fluctuations, Δ*E*, Δ*Z*, of the structural units that form the melt. In our previous works, the model has been applied to investigate the viscosity of many kinds of glass forming liquids such as covalent, ionic, molecular, metallic, and polymeric materials [24,25,26,27]. It has been shown that the model reproduces experimental data well, and characterizes many kinds of glass forming liquids extending from strong to fragile systems. From the theoretical side, it has also been shown that the viscous flow is accompanied by cooperatively rearranging movements of the structural units and occurs by breaking selectively weaker parts of the bonds [24,28]. This notion of the viscous flow is closely related to the well-known concept to explain the glass transition phenomena, the “cooperatively rearranging region (CRR)” as proposed by Adam and Gibbs [29]. Thus, our model of the thermally activated viscous flow, provides further understanding of the structural relaxation, in addition to other well-known theories such as the configurational entropy theory [29,30,31], the free volume theory [32,33], and to the picture obtained from the activated volume [34], *etc*.

In this article, it is shown that under certain conditions, the BSCNF model reproduces exactly the same viscosity behavior as the VFT equation [25,26,35]. The prerequisite is that the normalized bond strength fluctuation |Δ*E*|/*E*_0_ equals the normalized coordination number fluctuation |Δ*Z*|/*Z*_0_. This condition makes it possible to directly connect the parameters of the VFT equation, such as the ideal glass transition temperature *T*_0_, to the parameters of the BSCNF model, which contain microscopic information related to bonding connectivity among the constituent elements. Thus, the BSCNF model incorporates the VFT relation. By choosing the best fitting parameters, it reproduces the experimental data better than the VFT equation. In this review, the correlation between the fragility index of various glass forming liquids and the stretched exponent of the Kohlrausch-Williams-Watts (KWW) relaxation function [36,37], is also discussed in terms of the cooperativity which is defined by the BSCNF model.

## 2. The BSCNF Model and the VFT Equation

Commonly, glasses are formed by quenching a liquid. In the course of lowering the temperature, the value of the viscosity increases drastically reaching approximately 10^12^ Pa **·** s at the glass transition temperature *T*_g_. At the microscopic scale, the constituent elements of the glasses are considered to form certain types of clusters or structural units. Such structural units are bound to others by a certain bond strength retaining its spatial random connectivity. Within the glass-forming liquid, thermally activated viscous flow occurs due to bond-breaking and bond-switching. In addition, it must be noted that it is not necessary to break all the bonds connecting to the nearest neighbor components when the thermally activated viscous flow occurs. Bond twisting may also result in the viscous flow by enrolling the movement of second or more distant components of the melt.

Based on this picture, a model for the temperature dependence of the viscosity, the Bond Strength—Coordination Number Fluctuation (BSCNF) model, has been proposed by one of the authors [23]. The BSCNF model is given by

(1) $$\eta =\frac{\eta_{0}}{\sqrt{1-Bx^{2}}}\text{exp}\left[\frac{Cx+Cx^{2}\left[\left\{\text{ln}\left(\frac{\eta_{T\text{g}}}{\eta_{0}}\right)+\frac{1}{2}\text{ln}(1-B)\right\}\frac{\left(1-B\right)}{C}-1\right]}{1-Bx^{2}}\right]\text{,}$$

where

(2) $$B=\frac{{\left(\text{Δ}E\right)}^{2}{\left(\text{Δ}Z\right)}^{2}}{R^{2}{T_{\text{g}}}^{2}}\text{, and }C=\frac{E_{0}Z_{0}}{RT_{\text{g}}}\text{.}$$

Here, *x* is the inverse temperature normalized by *T*_g_, *x* = *T*_g_/*T*. *R* is the gas constant. *η*_0_ and *η_T_*_g_ are the viscosity at the high temperature limit and at the glass transition temperature, respectively. The fitting parameters *B* and *C* defined in Equation 2 have the following intuitive meanings: *C* gives the mean total binding energy per structural unit and *B* gives the degree of its fluctuations among the structural units against the thermal disturbance at *T*_g_. Regarding the number of parameters, the BSCNF model given in Equation 1 has five fitting parameters, namely, *B*, *C*, *η_T_*_g_, *η*_0_, and *T*_g_. However, it must be noted that the effect of *T*_g_ is embodied in *B* and *C* as given in Equation 2. Thus, in the case where the viscosity is plotted in the Angell’s plot, the BSCNF model given in Equation 1 has four fitting parameters, *i.e*., *B*, *C*, *η_T_*_g_, and *η*_0_.

**Figure 1:**
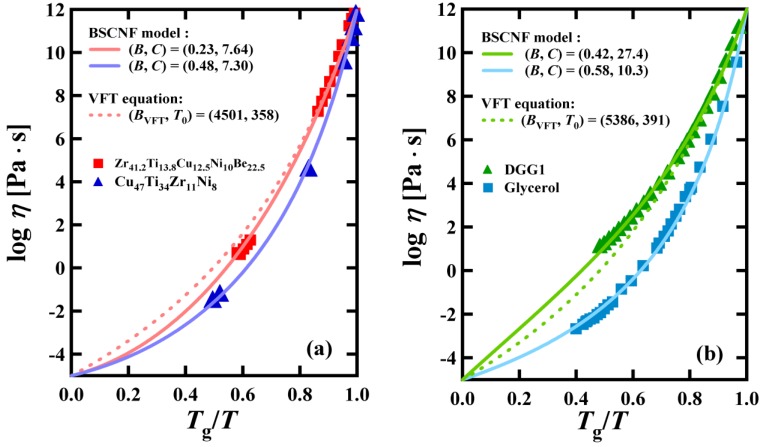
(a) Temperature dependence of the viscosity in bulk metallic glass forming liquids (Zr_41_Ti_13.8_Cu_12.5_Ni_10_Be_22.5_ and Cu_47_Ti_34_Zr_11_Ni_8_); and (b) DGG1 (Soda lime silicate glass) and Glycerol. The data shown with the symbols are taken from the reference [38] in (a) and reference [39] in (b). The full lines are reproduced by the BSCNF model, and the dashed lines are by the VFT equation given in Equation 5.

Figure 1 shows some applications of the BSCNF model. The viscosity of bulk metallic glass forming liquids such as Zr_41_Ti_13.8_Cu_12.5_Ni_10_Be_22.5_ and Cu_47_Ti_34_Zr_11_Ni_8_, and other type of materials such as DGG1 (a kind of soda lime silicate glass) and Glycerol, are shown in Figure 1 (a) and (b), respectively. As can be seen in these figures, the BSCNF model reproduces the viscosity data better than the VFT equation.

**Figure 2:**
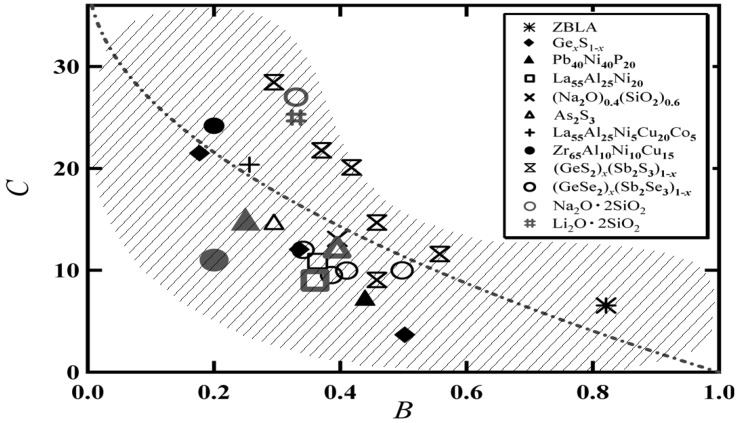
Mapping of different kinds of glass-forming liquids in the *B*-*C* space. The dashed curve indicates Equation 3 for the case of *γ* = 1 with *η_T_*_g_ = 10^12^ Pa **·** s and *η*_0_ = 10^ −5^ Pa **·** s. The correlation between *B* and *C* that has been suggested previously [24] is shown by shaded area.

According to the BSCNF model, glass forming liquids are characterized by the set of parameters *B* and *C* [23,24]. Figure 2 shows that many kinds of glass forming materials, including covalent, metallic, and molecular glassy systems, are characterized in a mapping plotted in the *B*-*C* space. From this figure, we note an interesting trend. Strong system is characterized by a large value of *C* and a small value of *B*. While, fragile system is characterized by a small value of *C* and a large value of *B*. In this manner, the BSCNF model can characterize any kind of glass forming material in terms of *B* and *C*, or, *E*_0_, *Z*_0_, Δ*E*, and Δ*Z*. It is expected that the characteristics of glass forming materials are reflected through these quantities. Furthermore, by studying the trend, we note that there is a correlation between *B* and *C*, which has been suggested in our previous work [24]. Such a correlation is shown by the shaded area in Figure 2. It has also been found that an analytical relation, reproducing this correlation between *B* and *C*, can be derived from the BSCNF model [28]. The relation is given by

(3) $$C=\frac{2\gamma (1-B)}{2\gamma +\sqrt{B}(1+\gamma^{2})}\left\{\text{ln}\left(\frac{\eta_{Tg}}{\eta_{0}}\right)+\frac{1}{2}\text{ln}(1-B)\right\}\text{,}$$

where

(4) $$\gamma =\frac{\left|\text{Δ}E\right|/E_{0}}{\left|\text{Δ}Z\right|/Z_{0}}.$$

Note that *γ* gives the ratio of the normalized bond strength fluctuation to the normalized coordination number fluctuation. The dashed line in Figure 2 shows the behavior given by Equation 3 for the case of *γ* = 1 with *η_T_*_g_ = 10^12^ Pa **·** s and *η*_0_ = 10^ −5^ Pa **·** s. In one of our previous works, the composition and temperature dependence of the viscosity in Cu*_x_*(As_2_Se_3_)_1−*x*_ (*x* ≤ 0.20) was discussed [28]. There, it was shown that in this system, the ratio of the fluctuations *γ* can take relatively large values, *γ* ≈ 15. The result suggests that for the Cu-As-Se system, there is a strong composition dependence in the bond strength fluctuation and a weak dependence in the coordination number fluctuation and fragility.

Furthermore, we have compared and discussed the interrelation between the VFT equation and the BSCNF model [25,26]. It has been found that in the case of *γ* = 1, the viscosity behavior described by Equation 1 with the set of parameters (*B*, *C*) obeying the relation Equation 3, perfectly follows the behavior described by the VFT equation,

(5) $$\text{log}\eta =A_{\text{VFT}}+\frac{B_{\text{VFT}}}{T-T_{0}}\text{,}$$

where *A*_VFT_ is the logarithm of the viscosity at the high temperature limit, *A*_VFT_ = log *η*_0_, and *B*_VFT_, *T*_0_ are the free fitting parameters of the VFT equation. It is considered that at the ideal glass transition temperature *T*_0_, which is also called “Vogel temperature”, the movement of the atoms is totally frozen. In the VFT equation, the number of fitting parameters is three, namely, *B*_VFT_, *T*_0_, and *η*_0_. One of the reasons that the BSCNF model reproduces the experimental data better than the VFT equation, as shown in Figure 1, is due to the difference in the number of free parameters. Here, it should be noted that for the case of *γ* = 1, the number of free parameters of the BSCNF model reduces from four to three, *B*, *η_T_*_g_, and *η*_0_, because *C* and *B* are connected mutually through Equation 3.

At the glass transition temperature, the VFT equation given by Equation 5 reduces to

(6) $$\text{ln}\left(\frac{\eta_{Tg}}{\eta_{0}}\right)=\frac{\text{ln}(10)\left(\frac{B_{\text{VFT}}}{T_{g}}\right)}{1-\left(\frac{T_{0}}{T_{g}}\right)}\text{.}$$

In Table 1, the key parameters for 38 kinds of oxide glass forming materials are indicated. In order to check the exact fitting between the VFT equation and the BSCNF model, in this analysis, we used the collection of fitting parameters by the VFT equation given in reference [4], where the values of *B*_VFT_, *T*_0_, *T*_g_, and the fragility index *m* for various oxide glass forming materials are provided. The numerical values of both, ln(*η_T_*_g_/ *η*_0_) calculated by Equation 6, and the best fitting parameters (B^*^, C^*^) determined by the BSCNF model, are also indicated in Table 1. 

The fragility index *m* is defined as *m* = d log *η*/d (*T*_g_/*T*)|*_T_*_=*T*g_ [21]. From the BSCNF model of the viscosity given in Equation 1, we obtain the fragility index *m* [23,24,25,26].

(7) $$m=\frac{1}{\text{ln}(10)}\left\{\frac{B-C+2\left[\text{ln}\left(\frac{\eta_{T\text{g}}}{\eta_{0}}\right)+\frac{1}{2}\text{ln}(1-B)\right]}{1-B}\right\}\text{.}$$

On the other hand, from the VFT equation given in Equation 5, we obtain another fragility expression,

(8) $$m=\frac{\left(\frac{B_{\text{VFT}}}{T_{\text{g}}}\right)}{{\left\{1-\left(\frac{T_{0}}{T_{\text{g}}}\right)\right\}}^{2}}.$$

**Table 1:** Parameters of various oxide glass forming materials. Data of *B*_VFT_, *T*_0_, *T*_g_, and *m* are taken from reference [4]. The values of ln(*η_T_*_g_/*η*_0_), and the best fitted parameters (B^*^, C^*^), are calculated from Equations 1, 3 and 7 under the condition that Equation 3 satisfies *γ* = 1.

No. Material	*B*_VFT _(K)	*T*_0_ (K)	*T*_g_ (K)	*m*	ln(*η_T_*_g_/*η*_0_)	(B^*^, C^*^)
**1.** SiO_2_	21,254	139	1,450	17.9	37.3	(0.01, 3.8)
**2.** Li_2_O·SiO_2_	5,744	276	593	33.9	41.7	(0.22, 2.3)
**3.** Li_2_O·2SiO_2_	5,752	380	727	34.7	38.2	(0.27, 18.2)
**4.** Li_2_O·3SiO_2_	8,218	255	734	26.3	39.5	(0.12, 25.7)
**5.** Na_2_O·SiO_2_	4,999	395	687	40.3	39.4	(0.33, 16.7)
**6.** Na_2_O·2SiO_2_	5,538	393	728	35.9	38.1	(0.23, 17.5)
**7.** Na_2_O·3SiO_2_	7,484	287	743	26.7	37.8	(0.15, 23.2)
**8.** Na_2_O·4SiO_2_	7,618	323	765	29.8	39.7	(0.18, 22.9)
**9.** K_2_O·SiO_2_	4,395	416	675	44.2	39.1	(0.38, 14.9)
**10.** K_2_O·2SiO_2_	7,461	333	768	30.3	39.5	(0.19, 22.3)
**11.** K_2_O·3SiO_2_	8,334	253	760	24.6	37.9	(0.11, 25.3)
**12.** K_2_O·4SiO_2_	8,471	255	766	24.8	38.2	(0.11, 25.5)
**13.** Na_2_O·Al_2_O_3_·6SiO_2_	12,281	347	1,087	24.4	38.2	(0.10, 26.0)
**14.** CaO·MgO·2SiO_2_	4,826	710	995	59.1	39.0	(0.51, 11.1)
**15.** CaO·Al_2_O_3_·2SiO_2_	5,802	785	1,113	60.0	40.7	(0.50, 11.9)
**16.** 2MgO·2Al_2_O_3_·5SiO_2_	8,244	583	1,088	35.2	37.6	(0.29, 17.4)
**17.** 15.45Na_2_O·12.81CaO·71.74SiO_2_	6,785	421	819	35.1	39.3	(0.26, 19.0)
**18.** 2BaO·TiO_2_·2SiO_2_	3,896	750	983	70.5	38.5	(0.58, 9.0)
**19.** PbO·SiO_2_	3,690	454	673	51.8	38.8	(0.46, 12.5)
**20.** PbO·2SiO_2_	6,001	390	749	34.9	38.5	(0.27, 18.4)
**21.** 2PbO·SiO_2_	2,496	473	613	78.1	41.1	(0.60, 9.2)
**22.** B_2_O_3_	4,695	252	540	30.6	37.5	(0.22, 20.0)
**23.** Li_2_O·B_2_O_3_	2,557	542	693	77.7	39.0	(0.61, 8.4)
**24.** Li_2_O·2B_2_O_3_	2,497	616	763	88.2	39.1	(0.65, 7.4)
**25.** Li_2_O·3B_2_O_3_	2,850	598	768	76.6	38.8	(0.61, 8.4)
**26.** Li_2_O·4B_2_O_3_	2,908	579	751	73.8	38.9	(0.60, 8.8)
**27.** Na_2_O·2B_2_O_3_	2,405	600	748	82.1	37.4	(0.65, 7.3)
**28.** Na_2_O·3B_2_O_3_	3,121	557	746	65.2	38.0	(0.56, 9.5)
**29.** Na_2_O·4B_2_O_3_	3,172	539	727	65.2	38.9	(0.55, 9.9)
**30.** K_2_O·2B_2_O_3_	2,888	520	705	59.5	36.0	(0.55, 9.3)
**31.** K_2_O·3B_2_O_3_	3,403	512	709	62.2	39.8	(0.52, 10.9)
**32.** K_2_O·4B_2_O_3_	3,588	463	691	47.7	36.2	(0.45, 11.9)
**33.** Cs_2_O·3B_2_O_3_	3,363	491	693	57.1	38.3	(0.50, 11.1)
**34.** BaO·2B_2_O_3_	3,262	619	810	72.4	39.3	(0.59, 9.2)
**35.** SrO·2B_2_O_3_	2,592	755	911	97.0	38.3	(0.69, 6.4)
**36.** PbO·B_2_O_3_	2,171	525	658	80.8	37.6	(0.64, 7.5)
**37.** PbO·2B_2_O_3_	3,020	545	738	59.8	36.0	(0.55, 9.3)
**38.** PbO·3B_2_O_3_	2,656	569	728	76.5	38.5	(0.61, 8.3)

## 3. Comparison between the BSCNF Model and the VFT Equation

**Figure 3:**
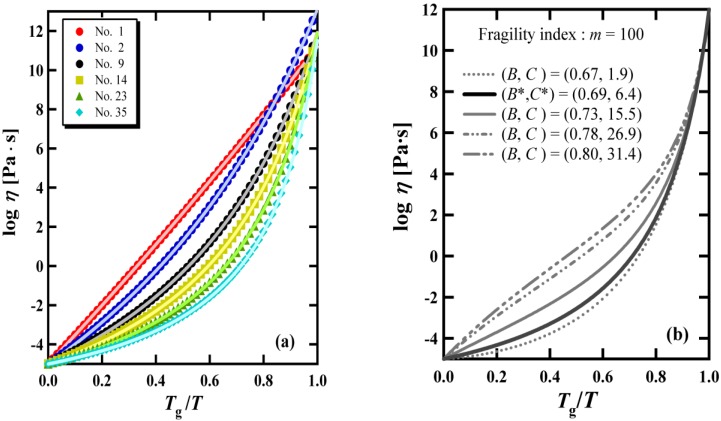
(a) The exact correspondence between the temperature dependence of viscosity described by the VFT equation (symbols) and that described by the BSCNF model (solid lines). The materials numbers are the same as given in Table 1. (b) The viscosity behaviors described by Equation 1 with five different values of (*B*, *C*). B^*^ and C^*^ indicate the value of *B* and *C* that perfectly reproduces the VFT behavior.

**Figure 4:**
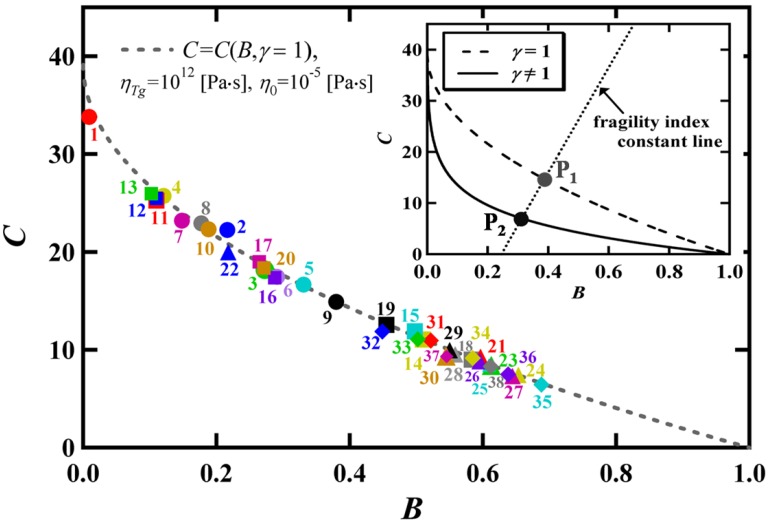
The viscosity described by Equation 1 using the set of parameters (B^*^, C^*^) that satisfy Equation 3 with *γ* = 1 corresponds exactly to that described by the VFT equation given in Equation 5. The *γ* dependence is illustrated in the inset.

Figure 3 (a) shows the complete correspondence of the viscosity behaviors reproduced by the VFT equation and the BSCNF model. In Figure 4, we can see that all the materials given in Table 1 are located on the curve *C* (*B*, *γ* = 1) described by Equation 3 in the *B*-*C* space. This result provides a physical interpretation to the VFT relation, from the BSCNF model’s point of view. Specifically, according to the BSCNF model, the glass forming liquids whose viscosity data are described by the VFT equation satisfies the following relation [35],

(9) $$\frac{\left|\text{Δ}E\right|}{E_{0}}=\frac{\left|\text{Δ}Z\right|}{Z_{0}}\text{.}$$

The physical meaning given by Equation 9 is clearer than that of the fitting parameters used in the VFT equation, because, for instance, the ideal glass transition temperature *T*_0_, which indicates the dynamical divergence in the temperature dependence of the viscosity or relaxation time, is not directly observed [18]. The quantities *E*_0_, *Z*_0_, and Δ*E*, Δ*Z* used in Equation 9 are in principle measurable quantities. It should be mentioned, however, that the theoretical justification of the derivation of the VFT equation from the BSCNF model, by imposing the condition given in Equation 9, remains to be solved. In Figure 2, it is shown that not all the materials are located on the curve of *C* (*B*, *γ* = 1). This result indicates that for different glass forming materials, the distribution of the connectivity among the structural units which is described by *E* and *Z* differs among the glassy materials. In the inset of Figure 4, it is shown that *C* (*B*, *γ* ≠ 1) deviates from *C* (*B*, *γ* = 1) When the set of values (*B*, *C*) at P_1_ is used, for instance, Equation 1 reproduces exactly the viscosity behavior described by the VFT equation as shown in Figure 3 (a). Thus, by changing (*B*, *C*), the BSCNF model can reproduce the experimental behavior better than the VFT equation. Such a set of values (*B*, *C*) is denoted schematically by P_2_ which is on the line of *C* (*B*, *γ* ≠ 1) in the inset. Figure 3 (b) shows the different viscosity behaviors described by Equation 1 with five different sets of (*B*, *C*). Here, (B^*^, C^*^) exactly reproduces the behavior given by the VFT equation. As noted above, by changing (*B*, *C*), the experimental data of the viscosity from strong to fragile glass forming systems are well reproduced. However, analogous to the case of the VFT equation [8,16,40], in some cases such as the van der Waals liquids [41] and metallic glass forming systems [42], the BSCNF model does not reproduce the experimental data over a wide temperature range. This is due to the fact that in the high temperature region above *T*_g_, a salient change in the transport properties emerges near the dynamical crossover temperature *T*_c_ as predicted by the widely discussed mode-coupling theory [5,8,13,14].

Using Equations 3, 6, and 8, the following relation that analytically connects the parameters of the VFT equation, *T*_0_, with that of the BSCNF model, *B* and *C*, is obtained,

(10) $$\frac{T_{0}}{T_{\text{g}}}=1-\frac{\left(\frac{1+\sqrt{B^{*}}}{1-B^{*}}\right)C^{*}-\frac{1}{2}\text{ln}(1-B^{*})}{\text{ln}(10)m}\text{,}$$

where B^*^ and C^*^ denote the values of *B* and *C* that satisfy Equation 3 in the case of *γ* = 1. It is noteworthy that B^*^ and C^*^ are calculated as a function of the fragility index *m*. In the inset of Figure 4, the point P_1_ is designated as (B^*^, C^*^) and denotes the intersection between the line of constant fragility index *m* with the curve *C* (*B*, *γ* = 1) given by Equation 3. In other words, if the value of fragility index *m* is given, the set of (B^*^, C^*^) can be determined uniquely by calculating the intersection. The applicability of Equation 10 has been discussed by applying it to some polymeric [25] and metallic materials [27]. The characteristic temperature ratio *T*_0_/*T*_g_, is related to the fragility [14,19,43,44,45]. While, by using the VFT equation, another expression for the characteristic temperature ratio *T*_0_/*T*_g_ is derived

(11) $$\frac{T_{0}}{T_{\text{g}}}=\frac{\text{ln}\left(\frac{\eta_{T_{g}}}{\eta_{0}}\right)}{D+\text{ln}\left(\frac{\eta_{T_{g}}}{\eta_{0}}\right)}\text{,}$$

or by using the fragility index *m*, we also have

(12) $$\frac{T_{0}}{T_{\text{g}}}=\left\{1+\frac{D}{2m}\right\}-\sqrt{\left(\frac{D}{m}\right)\left\{\frac{1}{4}\left(\frac{D}{m}\right)+1\right\}}\text{,}$$

where, *D* is the strength parameter defined as *D* = *B*_VFT_/*T*_0_ [45,46]. The expressions for *T*_0_/*T*_g_ given in the above equations have been used, for instance, in the analysis of pressure dependence of relaxation behavior of supercooled liquids [43]. Here, it must be noted that *T*_0_/*T*_g_ can be used as an index equivalent to the fragility, because the ratio takes values between 0 (the strongest) and 1 (the most fragile) [46]. The same statement applies to the expression given in Equation 10. For the case of strong systems, such as SiO_2_, B^*^ is nearly 0, and C^*^ takes approximately C^*^ ≈ 39.1. Thus, the right hand side of Equation 10 equals nearly 0, because for this system *m* ≈ 17. On the other hand, in more fragile systems, B^*^ and C^*^ take a larger and a smaller values, respectively. In such a case the value of *T*_0_/*T*_g_ given by Equation 10 approaches unity. Therefore, the information on bonding of the structural units is embodied in *T*_0_/*T*_g_, and is described in terms of the parameters of the BSCNF model by using the expression given in Equation 10. It was concluded in reference [18] that the prediction by the VFT equation with dynamic divergence at *T*_0_ lacks direct experimental evidences. Their experimental investigations indicate that a simple use of the VFT equation is not sufficient to fully understand the physics of glass transition phenomena behind structural relaxation. So far, quite a large number of studies regarding the VFT equation have been accumulated. By linking these large numbers of works with the result obtained from the BSCNF model, especially by using Equation 10, it is expected that further knowledge on structural relaxation in glass forming melt can be extracted.

## 4. Correlation between the Exponent of the KWW Function and the Fragility

Many physical quantities exhibit a universal feature in their structural relaxation behaviors which mainly involve the cooperative relaxation motions of the constituent elements toward the glass transition. To describe such a behavior, the Kohlrausch-Williams-Watts (KWW) function [36,37] has been widely used [5,8,10,17,33,40,47,48,49,50]. It is written as

(13) $$\phi_{\text{KWW}}(t)=\phi_{0}\text{exp}\left[{\left(\frac{t}{\tau }\right)}^{\beta_{\text{KWW}}}\right]\text{, }0<\beta_{\text{KWW}}\leq 1\text{,}$$

where $\phi_{0}$ is the value of the physical quantity at time *t* = 0. *β*_KWW_ is the exponent of the KWW function which is called “stretched exponent”, and *τ* is the structural relaxation time.

It is widely accepted that *β*_KWW_ gives the degree of many-body interactions among the constituent elements of structurally disordered materials [33,40,50]. It is known that in the case of *β*_KWW_ = 1, structural relaxation reduces to the Debye relaxation where all the elements of the glass exhibit a simple relaxation. For systems characterized by a small value of *β*_KWW_, on the other hand, many modes of relaxation are present, leading to non-linear relaxation [10,33]. It is also known that *β*_KWW_ is related to the fragility and that the non-linear relaxation behaviors are related to the α-relaxation described by the VFT equation [10,21].

According to Vilgis [51], the stretched exponent *β*_KWW_ and the strength parameter *D* are mutually connected through the following relation

(14) $$\beta_{\text{KWW}}=1-\sqrt{\frac{{\left(T_{0}/T_{\text{g}}\right)}^{2}}{D}}.$$

Equation 14 is used to check the value of *β*_KWW_ with the VFT equation [45,52]. Using this relation together with Equation 8, the following relation between the stretched exponent *β*_KWW_, the characteristic temperature ratio* T*_0_/*T*_g_, and the fragility index *m* is obtained,

(15) $$\beta_{\text{KWW}}=1-\frac{\left(T_{0}/T_{\text{g}}\right)}{1-(T_{0}/T_{\text{g}})}\sqrt{\frac{\left(T_{0}/T_{\text{g}}\right)}{m}}.$$

**Figure 5:**
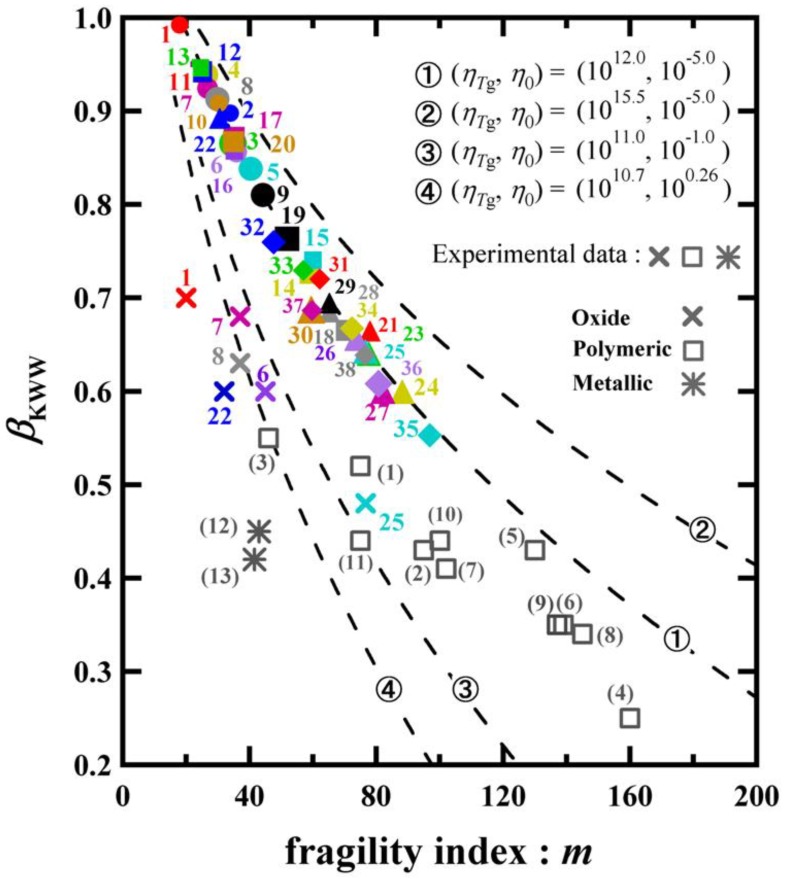
Relation between the exponent of KWW function and the fragility index. The dashed curves are calculated from Equation 15 with *T*_0_/*T*_g_ given in Equation 10 by varying the values of *η_T_*_g_ and *η*_0_. The colored symbols are the same as those used in Figure 4. Actual values determined by experiments are also indicated. The experimental data shown in this figure are given in Table 2.

In Figure 5, the relation between the exponent of the KWW function and the fragility index is shown. The symbols used in Figure 5 are the same as those used in Figure 4. The values of *β*_KWW_ are calculated from Equation 15. The dashed curves in Figure 5 are calculated from Equation 15 with* T*_0_/*T*_g_ given in Equation 10. We can see that all values of *β*_KWW_ for the materials indicated in Table 1 follow the theoretical curve when *η_T_*_g_ = 10^12^ Pa **·** s and *η*_0_ = 10^−5^ Pa **·** s are used. A correlation between the fragility index *m* and *β*_KWW_ has been observed in many kinds of glass-forming liquids [17,21,48,49]. It is expressed as *m* = *C*_1_ − *C*_2_*β*_KWW_, where *C*_1_ and *C*_2_ are constants [21]. This phenomenological relation is analogous to our result shown in Figure 5. It must be noted however that for some glassy materials, the data measured do not necessarily follow the theoretical curve calculated with *η_T_*_g_ = 10^12^ Pa **·** s and *η*_0_ = 10^ −5^ Pa **·** s. In Figure 5, other theoretical curves calculated by using different values of *η_T_*_g_ and *η*_0_ are also shown. The same approach has been employed for many types of bulk metallic systems [27]. Although the validity of Equation 10 is restricted to the case of *γ* = 1, the result of Figure 5 provides a sound physical meaning of *β*_KWW_ in respect to the BSCNF model.

Previously, a quantity defined as *N*_B_ = *E_η_* /(*E*_0_*Z*_0_) which gives the number of structural units involved in the thermally activated viscous flow has been introduced in the framework of the BSCNF model [24]. Here, *E_η_* denotes the activation energy for the viscous flow. It has been shown that *N*_B_ can be expressed analytically by *B* and *C*, *N*_B_ (*B*, *C*) at *T*_g_, and the value of *N*_B_ increases with the increase in the fragility index *m* [28]. From the results, it was suggested that the thermally activated viscous flow occurs when the weaker parts of bonds are selectively broken. This observation indicates that systems with a smaller value of *β*_KWW_ correspond to systems that have a larger value of *N*_B_. In this regard, some researchers have reported similar ideas. For instance, Park *et al*. have mentioned that the non-exponentiality given by *β*_KWW_ is directly affected by the intermolecular cooperativity, resulting in the increase of the domain size of the glass-forming alcohols [10]. Similarly, Rault has indicated that the connection between cooperative motion of α- relaxation and *β*_KWW_ leads to the VFT law [53]. This picture of the cooperativity gained through the quantity *N*_B_ is also in harmony with the concepts proposed by others such as the “cooperativity of atomic migrations” given by Wang and Fecht [54], and the “supercooled liquid as an elastic medium” given by Trachenko [55,56], *etc*. Our view of the cooperativity gives the same result and provides an alternative view to understand the structural relaxation, that is, in terms of the structural units which is determined by the bonding nature. All these results indicate that the BSCNF model could be an alternative to the VFT equation which has been widely used in the analysis of the temperature dependence of the viscosity. The BSCNF model provides a clearer physical picture and microscopic information on bonding and cooperativity of the constituent elements within the viscous liquids.

**Table 2:** Experimental values of the stretched exponent *β*_KWW_ and the fragility index *m* for some materials such as oxide, polymeric, and metallic systems.

	Materials	*m*	*β*_KWW_	Reference
**1.**	SiO_2_	20	0.70	[21]
**6.**	Na_2_O**·**2SiO_2_	45	0.60	
**7.**	Na_2_O**·**3SiO_2_	37	0.68	
**8.**	Na_2_O**·**4SiO_2_	37	0.63	
**22.**	B_2_O_3_	32	0.60	
				
**25.**	Li_2_O**·**3 B_2_O_3_	77	0.48	[47]
				
**(1)**	Poly(propylene glycol)	75	0.52	[17]
**(2)**	Poly(vinyl acetate)	95	0.43	
				
**(3)**	Polyisobutylene	46	0.55	[57]
**(4)**	Polyvinyle chloride	160	0.25	
**(5)**	Polyvinyl acetate	130	0.43	
**(6)**	Polystyrene	139	0.35	
**(7)**	Polymethyl acrylate	102	0.41	
				
**(8)**	Poly(methylmethacrylate)	145	0.34	[21]
**(9)**	Polypropylene	137	0.35	
**(10)**	Poly(methylphenysiloxane)	100	0.44	
**(11)**	Poly(vinylmethylether)	75	0.44	
				
**(12)**	Zr_65_Al_7.5_Cu_17.5_ Ni_10_	43	0.45	[48]
**(13)**	Pd_40_Ni_40_P_40_	42	0.42	

## 5. Conclusions

In this paper, the BSCNF model has been reviewed. In particular, it was shown that in the case where the magnitudes of energy and coordination number fluctuations of the structural units are equal, the viscosity behavior described by the BSCNF model corresponds perfectly to that described by the VFT equation. It was also shown that the characteristic temperatures ratio *T*_0_/*T*_g_ can be expressed in terms of the parameters of the BSCNF model, namely, the total bond strength, average coordination number and their fluctuations of the structural units, leading to a new way to understand the cooperativity of the structural relaxation in supercooled liquids. Furthermore, by connecting the BSCNF model with another model given by Vilgis, a theoretical relation that correlates the stretched exponent of the KWW function *β*_KWW_, and the fragility index *m* has been obtained.
